# Black Ginseng and Its Saponins: Preparation, Phytochemistry and Pharmacological Effects

**DOI:** 10.3390/molecules24101856

**Published:** 2019-05-14

**Authors:** Ahmed M. Metwaly, Zhu Lianlian, Huang Luqi, Dou Deqiang

**Affiliations:** 1 Liaoning University of Traditional Chinese Medicine, 77 Life one Road, DD port, Dalian Economic and Technical Development Zone, Dalian 116600, China; ametwaly@azhar.edu.eg (A.M.M.); lianlianzhu1991@126.com (Z.L.); 2 Department of Pharmacognosy, Faculty of Pharmacy, Al-Azhar University, Cairo 11884, Egypt; 3 National Resource Center for Chinese Materia Medica, China Academy of Chinese Medical Sciences, 16 Mennei South street, Dong-Cheng District, Beijing 100700, China

**Keywords:** black ginseng, steaming and drying, transformed ginsenosides, anticancer, anti-inflammatory

## Abstract

Black ginseng is a type of processed ginseng that is prepared from white or red ginseng by steaming and drying several times. This process causes extensive changes in types and amounts of secondary metabolites. The chief secondary metabolites in ginseng are ginsenosides (dammarane-type triterpene saponins), which transform into less polar ginsenosides in black ginseng by steaming. In addition, apparent changes happen to other secondary metabolites such as the increase in the contents of phenolic compounds, reducing sugars and acidic polysaccharides in addition to the decrease in concentrations of free amino acids and total polysaccharides. Furthermore, the presence of some Maillard reaction products like maltol was also engaged. These obvious chemical changes were associated with a noticeable superiority for black ginseng over white and red ginseng in most of the comparative biological studies. This review article is an attempt to illustrate different methods of preparation of black ginseng, major chemical changes of saponins and other constituents after steaming as well as the reported biological activities of black ginseng, its major saponins and other metabolites.

## 1. Introduction

Ginseng is the roots and rhizomes of *Panax ginseng* Mey., belonging to the perennial plants of genus Panax and family Araliaceae. The most commonly used species are: *Panax ginseng* (Asian ginseng), *Panax quinquefolius* (American ginseng) and *Panax notoginseng* (Chinese notoginseng or Sanqi) [1]. Ginseng has been used in China for more than 4000 years as a tonic and restorative, promoting health, treating hemorrhage, impotence, anorexia and infections. Modern clinical studies proved various pharmacological effects for ginseng. For instance, the aphrodisiac and adaptogenic properties of ginseng were reported to result from its effects on the hypothalamic–pituitary–adrenal axis, resulting in an elevation of corticotropin and corticosteroids levels in plasma [2]. Moreover, the immune enhancing activity was correlated to its ability to regulate different types of immune cells such as dendritic cells, macrophages, natural killer cells, B cells and T cells [3]. Induction of apoptosis, inhibition of angiogenesis of cancer cells in addition to inhibition of cell proliferation and immunosurveillance are reported mechanisms for the anticancer activities of *Panax ginseng* [4]. According to in vitro and in vivo results, ginseng could treat cardiovascular diseases through antioxidation properties, reduction of platelet adhesion, vasomotor regulation, enhancing lipid profiles, and influencing several ion channels [5]. What is more, ginseng exhibited treating effects in many central nervous system disorders such as Parkinson’s disease, Alzheimer’s disease, depression, cerebral ischemia as well as several other neurodevelopmental disorders. Ginseng could exert these activities through different pathways such as neuroprotection, synaptic plasticity regulation, decreasing neuroinflammatory processes and regulation of neurotransmitter release [6].

Ginseng has been used as a dietary supplement in several countries all over the world; it was among the top 10 selling herbal dietary supplements in the USA in 2003, for instance; Korean red ginseng (*P. ginseng*) has been used as a food in the forms of chocolate, granular tea, root slices preserved with honey, jelly and candies [7]. In addition, in 2012, ginseng aged less than 5 years was approved to be used as a food material in China, which increased its production and uses [8].

**Figure 1:**
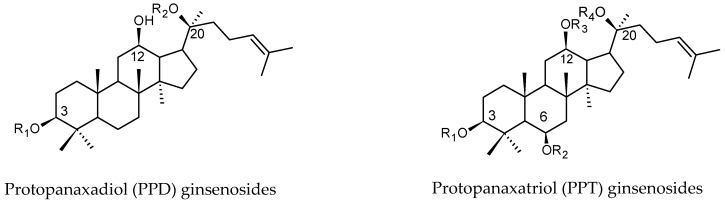
Major classes of Ginsenoside.

The main bioactive secondary metabolites in ginseng are ginsenosides, which belong to dammarane-type triterpene saponins with different sugar moieties attached at C-3 and C-20. Ginsenosides are named ‘Rx’: ‘R’ refers to the root and ‘x’ describes the ascending alphabetical order of their chromatographic polarity. Accordingly, Ra is the highest polar ginsenoside, while Rb is less polar than Ra [9]. As shown in Figure 1, ginsenosides can be classified into two major classes; (1) the protopanaxadiol (PPD) type in which the sugar units are attached to the β-OH at C-3 and/or C-20 (such as Ra_1_, Ra_2_, Ra_3_, Rb_1_, Rb_2_, R_c_, R_d_, Rg_3_, Rh_2_ and Rs_1_) and (2) the protopanaxatriol (PPT) type in which the sugar units are attached to the α- OH at C-6 and/or the β-OH at C-20 (such as Re, Rf, Rg, Rg_1_, Rg_2_ and Rh_1_). Moreover, some other ginsenosides, such as ocotillol group, have a five-membered epoxy ring at C-20 (for instance F11) and the oleanolic acid saponins (for example, Ro, C-IV and C-Ib) have also been isolated and identified [10]. The major ginsenosides in the white or (sun-dried) ginseng’s roots are Rg_1_, Re, Rb_1_, Rc, Rb_2_, Rb_3_ and Rd, which make up more than 70% of total ginsenosides [11]. In addition, some PPD or PPT-type ginsenosides with structure changes in side-chain were isolated and exhibited strong biological activities [12].

According to our previous research, the three generally-used species in *Panax* genus could be distinguished by the presence of some characteristic ginsenosides such as the notoginsenoside R_1_, which is a characteristic marker for *P. notoginseng,* while the ginsenoside Rf is a marker for *P. ginseng.* Furthermore, the ocotillol-type triterpene 24(R)-pseudo-ginsenoside F_11_ presents in high amounts in *P. quinquefolius* and in very minute amounts in *P. ginseng*, and hence a high ginsenoside Rf/24(R)-pseudo-ginsenoside F_11_ ratio (>700) clearly differentiates *P. ginseng* and *P. quinquefolius* [13]. Likewise, the ratio between Rb_1_ and Rg_1_ is a very clear marker as the high Rb_1_/Rg_1_ ratio (around 10 or greater) indicates *P. quinquefolius*, while low (1–3) indicates *P. ginseng* [14]. Additionally, the ginseng plant contains some other important secondary metabolites such as ginseng oils, phytosterol, carbohydrates, amino acids, peptides, vitamins, minerals, certain enzymes and phenolic compounds (caffeic acid, syringic acid, p-coumaric acid, ferulic acid and cinnamic acid) [15].

Due to black ginseng having originated and being distributed in Asia, the term black ginseng in this review refers to black *P. ginsing*, unless otherwise stated.

## 2. Preparation

Processing is an important step for the use of herbal medicines in traditional Chinese medicine (TCM). Traditionally, white, red and sugar ginseng were used in TCM. The white or (sun-dried) ginseng is produced by direct air-drying of the peeled roots and rhizomes without steaming, while if the roots have been steamed at around 100 °C before drying, they will get a red appearance and become what is known as red ginseng. Sugar ginseng is prepared by injection of sucrose water into the fresh ginseng to modify its flavor [16].

Black ginseng is usually developed from fresh or white ginseng steamed several times (usually 9 times) at 96 °C for 3 h, followed by hot air-drying at 50 °C for 24 h [17,18]. While the processing method of “steaming and drying 9 times” has always been used in TCM in the processing of Radix Rehmanniae [19], this method was rarely used for the processing of ginseng in the ancient TCM. Black ginseng was firstly prepared in South Korea then widely used in China and Southeast Asian countries. The optimum conditions for the preparation of black ginseng were determined to be steaming at 113.04 °C for 18 h and drying at 100 °C for 8.03 h; this method resulted in 0.75 mg/g ginsenoside Rg_3_, 3.24 mg/g polyphenol, 13.72 mg/g acidic polysaccharide and 0.26 part per billion (ppb) benzopyrene [20]. Other methods have been reported in the preparation of black ginseng, such as steaming the white ginseng three times at 120 °C for 30 min after soaking it in grape juice for 24 h, which was more effective in the transformation of ginsenoside Rg_3_ compared to the traditional method (got Rg_3_ approximately 18 times more than that in red ginseng) [21]. In addition, black ginseng was prepared by steaming white ginseng for 3 h, drying and steaming again for 6 h. In this procedure, the main ginsenosides were Rg_3_, Rk_1_ and Rg_5_ and the ginsenoside Rg_3_ was determined to be 11.48 mg/g of dried black ginseng [22]. The method of fermented black ginseng preparation was done by repeated steaming and drying of fresh ginseng before fermentation by incubation with Saccharomyces cerevisiae for 24 h [23]. On the other hand, flavored black ginseng was obtained by immersing white ginseng in garlic juice for 24 h, then putting it into the autoclave at 120 ℃ for 3 h, followed by air-drying in the oven at 60 ℃. In this procedure, the primary saponins in ginseng, such as ginsenoside Re, Rg_1_ and Rb_1_, almost completely transformed to the rare secondary saponins and aglycones such as ginsenoside Rg_3_, Rg_5_ and protopanaxadiol. In addition, non-reducing polysaccharides such as starch are completely degraded into smaller reducing sugar units [24]. Sun ginseng is a new type of processed ginseng prepared by steaming of fresh ginseng at 120 °C for 2 h using an autoclave. Accordingly, ginsenosides Rg_5_ and Rg_3_ were found to be the most transformed ginsenosides with concentrations of 19% and 39% of all ginsenosides, respectively [25]. The optimum conditions to prepare black ginseng with safe levels of benzo(a)pyrene were reported to be: Steaming at a temperature between 80 and 120 °C and drying at a temperature less than 50 °C [26].

To sum up, black ginseng can be processed through three different methods; the first one depends on the steaming and drying of fresh ginseng several times; the second method involves the steaming for one long time or for one short time under pressure (using an autoclave) and drying; the third method includes a fermentation process after the steaming and drying of ginseng. In addition, some flavors may be added to get flavored black ginseng, such as garlic and grape juice. The common chemical change of ginsenosides is the predominance of the ginsenoside Rg_3_. Recently, black ginseng was produced using cultivated ginseng in China, resulting in a product with Rg_3_ content of more than 1 mg/g [27].

## 3. Phytochemistry

**Figure 2:**
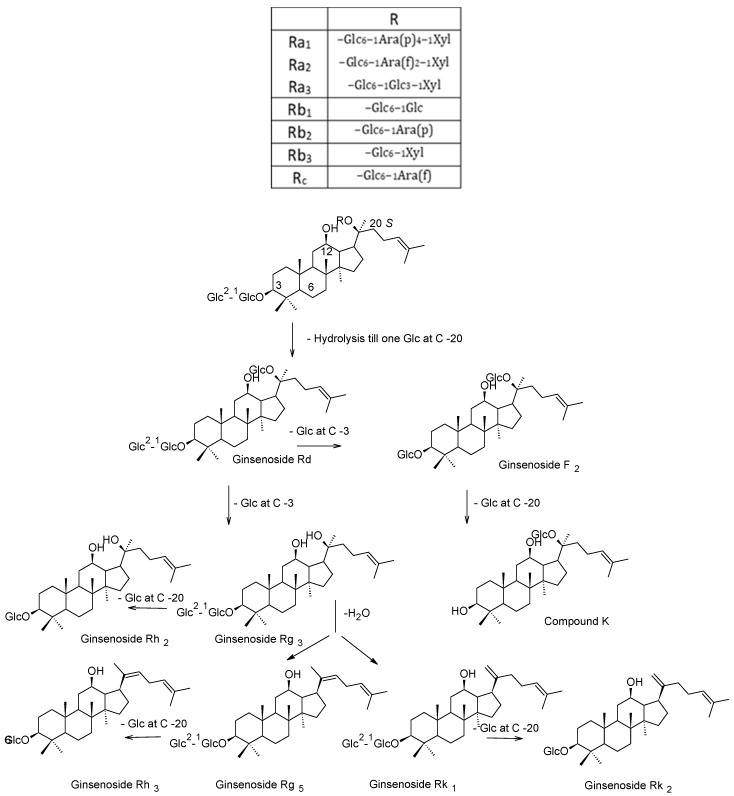
Pathways of chemical changes of protopanaxadiol (PPD)-type ginsenosides during black ginseng processing.

During the steaming process, ginseng secondary metabolites pass through different chemical reactions and transform into other forms. The polar ginsenosides transform into specific less-polar ginsenosides by hydrolysis, dehydration, decarboxylation and isomerization reactions at C-3, C-6 or C-20. The notable structural changes are the hydrolysis of sugar moieties at C-3, C-6 or C-20 and subsequent dehydration at C-20. These reactions took place with the major primary ginsenosides (Rb_1_, Rb_2_, Rc, Rd, Re and Rg_1_) as well as with other minor ginsenosides [28]. As shown in Figure 2, the ginsenosides Ra_1,_ Ra_2,_ Ra_3,_ Rb_1_, Rb_2_, Rb_3_, Rc and Rd are converted to the ginsenosides Rg_3_, F_2_, compound K and Rh_2_ in black ginseng through hydrolysis reactions for sugar moieties at C-3 and C-20. On the other side, the ginsenosides Rk_1_ and Rg_5_ are produced as a result of the dehydration reactions of Rg_3_ [11,18,29]. The R-epimers of Rg_2_, Rg_3_ and Rh_1_ were produced through isomerization of the corresponding ginsenosides as well as through addition reactions (selective attack of the hydroxyl group) for the dehydrated corresponding ginsenosides (Rk_1_ and Rg_5_ in case of 20(R)-Rg_3_) [17,30].

**Figure 3:**
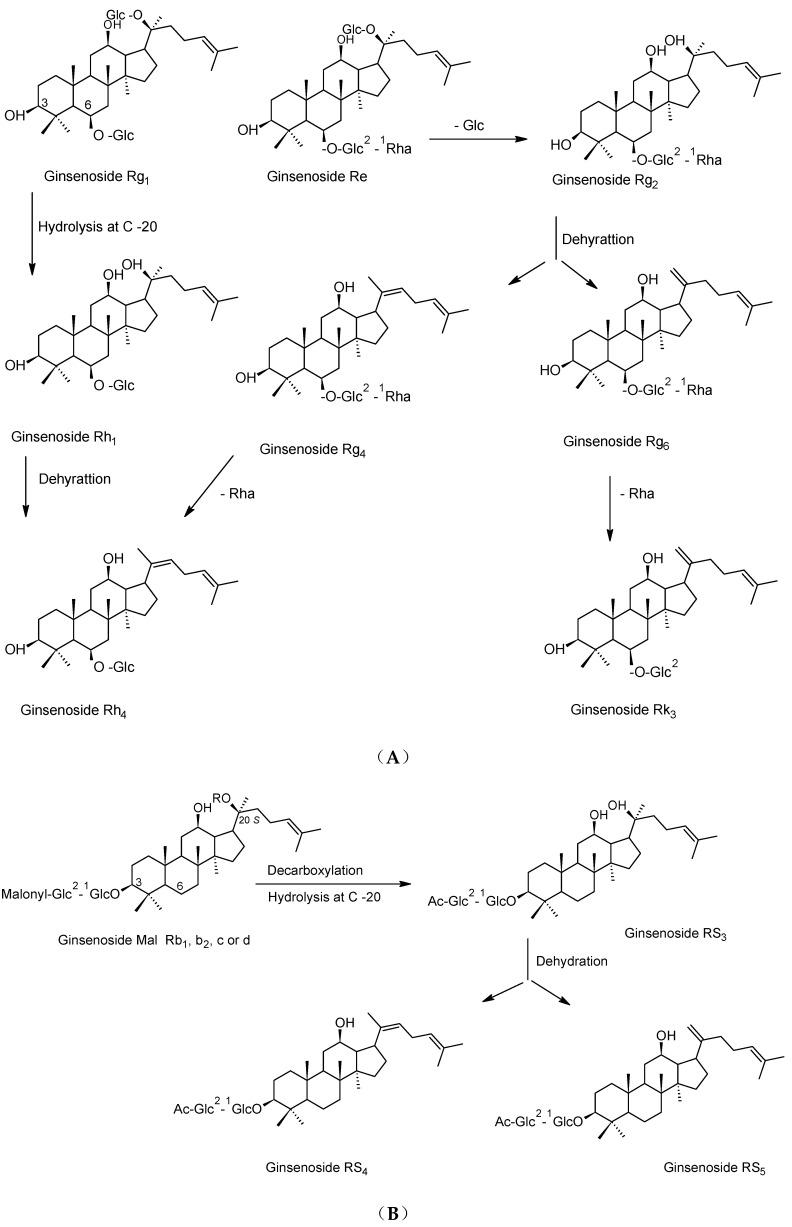
Pathways of chemical changes of some protopanaxatriol (PPT) (**A**) and PPD (**B**) type ginsenosides during black ginseng processing.

Rh_1_ and Rh_4_ were deduced to be generated from the ginsenoside Rg_1_ through hydrolysis and dehydration reactions (Figure 3A), respectively. Interestingly, four ginsenoside (Rg_6_, F_4_, Rk_3_ and Rh_4_) were produced from the ginsenoside Rg_2_ (Figure 3A) through hydrolysis and dehydration reactions [30]. The acetylated ginsenosides (20(S)-Rs_3_ and 20(R)-Rs_3_) were produced from the malonyl derivatives of Rb_1_, Rb_2_, Rc and Rd the by hydrolysis of glycosyl moiety at C-20 and decarboxylation of malonyl moiety attached to glycosyl linkage at C-3 (Figure 3B), 20(S)-Rs_3_ further underwent dehydration to generate Rs_4_ and Rs_5_ [29]. Generally, the steaming process causes an increase in the protopanaxadiol group to protopanaxatriol group ratio (PD/PT) from 1.9 to 8.4 in white and black ginseng, respectively [18]. In addition, it led to the production of some specific ginsenosides such as (20(S)-, 20(R)-Rg_3_, Rk_3_, Rh_4_, Rk_1_, Rg_5_, etc.) which are absent from white ginseng [30,31]. As black ginseng was subjected to much more steaming, the concentration of these ginsenosides is much higher than red ginseng.

Similarly, the steaming and drying processes led to some chemical changes in the other secondary metabolites. While steaming causes a decrease in the contents of polysaccharides from 29.1% in fresh ginseng to be only 11.1% in black ginseng [32], it causes an increase in reducing sugars and acidic polysaccharide contents. The increase of reducing sugars and acidic polysaccharide contents in black ginseng with percentages of 128% and 187.5%, respectively, comparing the white ginseng, was reported [17]. Similarly, the phenolic compounds content was increased more than threefold by steaming from 3.1 mg/g in white ginseng to be 10.6 mg/g in black ginseng [17]. In another comparative study, the phenolic contents of white ginseng and black ginseng roots of *Panax ginseng*, *P. notoginseng* and *P. quinquefolium* were evaluated to be 20.4 ± 0.90 mg/g, 17.12 ± 0.56 mg/g and 14.45 ± 0.13 mg/g in white ginseng roots and 34.3 ± 0.18, 44.15 ± 1.45, and 34.05 ± 2.03 mg/g in the black ginseng roots, respectively. The identified phenolics included ferulic, gentisic, cinnamic, syringic and p-hydroxybenzoic acids combined with arginine and maltose due to Maillard reaction [33]. Another study reported the increase of salicylic acid, vanillic acid and p-coumaric acid contents from 0.121, 0.404 and 0.522 mg/100g in white ginseng to 0.394, 0.628 and 0.737 mg/100g in black ginseng, respectively, because of the steaming process [34].

In fact, the black color of black ginseng is a result of a chemical reaction named Maillard reaction, which is a chemical reaction between reducing sugars and amino acids resulting in glycosylamines and or ketosamines [35]. The reported Maillard reaction products in ginseng due to steaming included: Argin-maltose [20] Arg-fru-glc, Arg-fru and maltol-3-O-β-D-glucoside in addition to maltol (2-Methyl-3-Hydroxy-4-Pyrone) which increased from 2.598 mg/100g in white ginseng to 94.007 mg/100g by steaming [34]. The content of maltol was reported to be much higher in black ginseng than red and white ginseng [36,37]. Melanoidins are a group of the Maillard reaction products and were produced by the combination of 5-hydroxymethyl-2-furaldehyde (5-HMF) with carbohydrates, amino acids and proteins, while 5-HMF are degradation products of carbohydrates because of the steaming and drying process. The content of 5-HMF increased gradually from 0 mg/g of white ginseng to 3.58 mg/g of black ginseng [30].

A significant decrease of free amino acids from 17.9 mg/g in white ginseng to 2.79 mg/g after steaming has been reported [38]. Another study outlined the decrease from 9.1% in fresh ginseng to 3.1% in black ginseng [32]. Arginine’s content (the most predominant amino acid in ginseng) was reduced from 10.4 to 1.38 mg/g and β-N-oxalyl-L-a,b-diaminopropionic acid (β-ODAP), a famous neurotoxin, was decreased also by 92.9%. The decrease of amino acid content in black ginseng is believed to be a result of Maillard reaction due to the detection of increased levels of Maillard reaction products [38]. These results have been authenticated by a recent study using multiple ultra-performance liquid chromatography with mass spectrometry (UPLC-MS) assay methods and which proved the decrease of amino acid contents during the steaming process. The concentrations of 29 amino acids were less in red ginseng than white ginseng and were the least in black ginseng [39].

**Figure 4:**
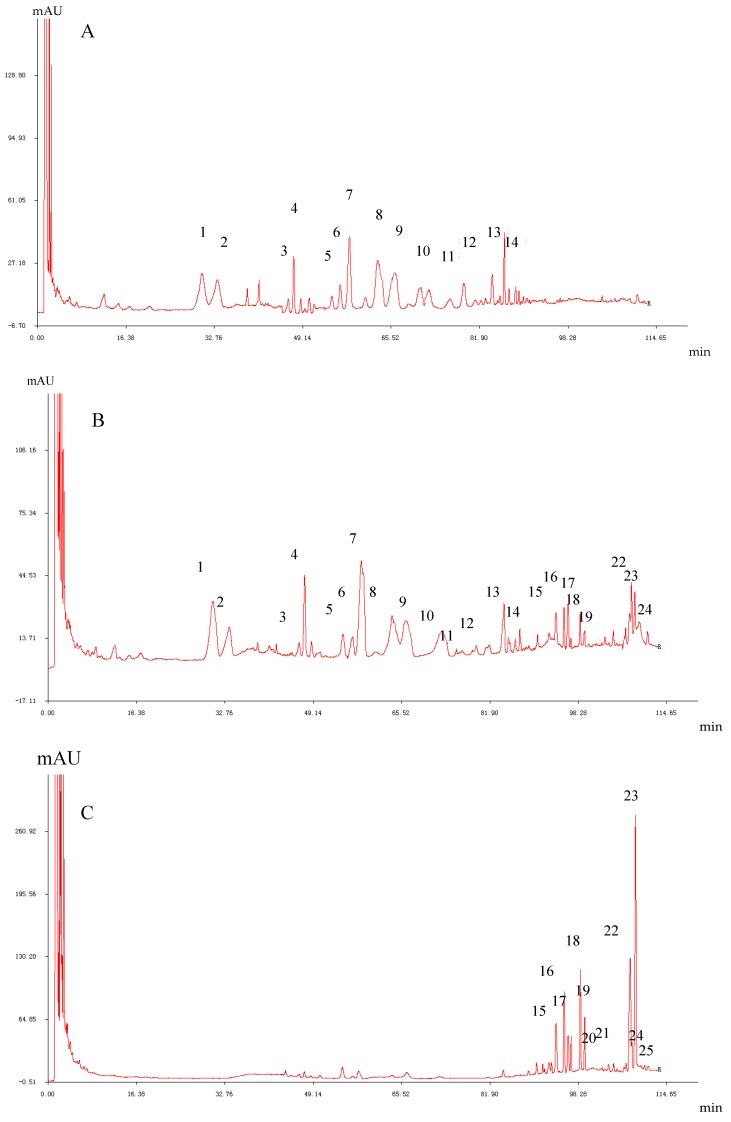
HPLC fingerprints of ginsenosides in white *P. ginseng* (**A**), red *P. ginseng* (**B**) and black *P. ginseng* (**C**). Note: 1-Rg_1_; 2-Re; 4-Rf; 5-(S)-Rg_2_; 6-(R)-Rg_2_; 7-Rb_1_; 8-Rc; 9-Ro; 11-Rb_2_; 12-Rb_3_; 13-Rd; 18-(S)-Rg_3_; 19-(R)-Rg_3_; 20-(S)-Rs_3_; 21-(R)-Rs_3_; 22-Rk_1_; 23-Rg_5_; 24-(S)-Rh_2_; 25-(R)-Rh_2_.

**Figure 5:**
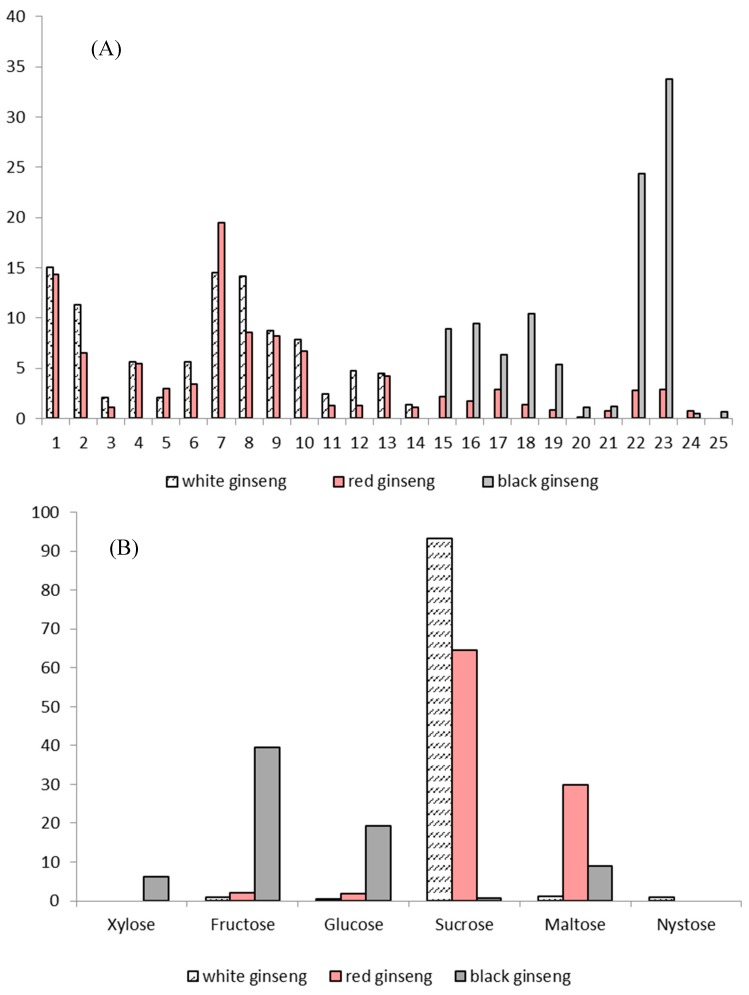
The relative percentage of individual ginsenosides (**A**) and oligosaccharides (**B**) in white *P. ginseng*, red *P. ginseng* and black *P. ginseng*. Note: 1-Rg_1_; 2-Re; 4-Rf; 5-(S)-Rg_2_; 6-(R)-Rg_2_; 7-Rb_1_; 8-Rc; 9-Ro; 11-Rb_2_; 12-Rb_3_; 13-Rd; 18-(S)-Rg_3_; 19-(R)-Rg_3_; 20-(S)-Rs_3_; 21-(R)-Rs_3_; 22-Rk_1_; 23-Rg_5_; 24-(S)-Rh_2_; 25-(R)-Rh_2_.

Figure 4 shows the high performance liquid chromatography (HPLC) fingerprints of ginsenosides in white *P. ginseng* (Figure 4A), red *P. ginseng* (Figure 4B) and black *P. ginseng* (Figure 4C) while Figure 5 explains the relative percentage of individual ginsenosides (Figure 5A) and oligosaccharides (Figure 5B) in white *P. ginseng*, red *P. ginseng* and black *P. ginseng* according to the published results of a recent comparative analytical study [27].

On the other hand, benzo(a)pyrene hydrocarbon was detected only in the black ginseng with a content of 0.17 μg/kg [17] and in another experiment it was 0.26 ppb [20]. Benzo(a)pyrene is a polycyclic aromatic hydrocarbon produced as a byproduct of the incomplete burning of organic materials. It is a potent carcinogenic and is listed as Group 1 carcinogens by the International Agency for Research on Cancer [40]. According to the Korea Food and Drug Administration (KFDA), the maximum acceptable level for benzo(a)pyrene in any food product is 5.0 μg/kg [17]. Accordingly, although the presence of benzo(a)pyrene hydrocarbon in black ginseng is reported, it is still in very minute amounts and much lower than the accepted limit.

## 4. Pharmacological Studies

During the past decade, there was considerable interest in investigating black ginseng’s pharmacological actions using different biochemical, pharmacological and molecular biological techniques. The examined pharmacological activities included anticancer, hepatoprotective, antidiabetic, antioxidant and general tonic activities besides its effect on the central nervous and immune systems. Some of these reports were carried out to compare the biological activity of black ginseng with white and/or red ginseng. While most of these reports were designed to figure out the pharmacological potential of black ginseng in addition to its mechanism of action. Furthermore, there is extensive literature involving the biological activities of the major transformed ginsenosides, such as Rg_3_, Rk_3_, Rh_2_, Rk_1_ and Rg_5_, which are present in black ginseng in much higher concentrations than red ginseng and are totally absent in white ginseng. Additionally, the amounts of polyphenols, acidic polysaccharides and Maillard reaction products increase considerably by the steaming process to be in the highest concentrations in black ginseng. In the few next pages we will discuss some published biological studies reporting black ginseng total extract and pure fractions as well as the transformed ginsenosides and other highly concentrated metabolites.

### 4.1. Anticarcinogenic Effects

Black ginseng and its major ginsenosides exerted promising in vitro and in vivo anticarcinogenic effects against several cancer types through different mechanisms of action. Some of them showed strong clinical results and some others were used as anti-cancer products in the market.

Black ginseng’s crude saponin fraction exerted stronger in vitro cytotoxic activities than red ginseng against ACFIN, HCT-15 and PC-3 cell lines with IC_50_ values ranging from 60.3–90.8 μg/mL [22]. In another comparative study, black ginseng extract exhibited cytotoxic activity against colon 26-M3.1 carcinoma cell line with an IC_50_ value of 800 μg/mL, while it was 2000 μg/mL for white ginseng [41]. In addition, black ginseng extract could inhibit basic fibroblast growth factor (bFGF)-induced endothelial cell proliferation and migration dose-dependently through its ability to inhibit the angiogenesis process [42]. Furthermore, breast cancer MCF-7 cell lines proliferation was inhibited by black ginseng due to cell cycle arrest induction in the G0/G1 phase in a dose-dependent manner [43].

Black ginseng exhibited promising anticancer activities in different in vivo studies such as tumor inhibition in H_22_ tumor-bearing mice dose-dependently via immune function improvement and tumor cell apoptosis induction [24]. Moreover, it performed in vivo anticancer activities against hepatocellular carcinoma as it reduced the size and the volume of an HepG2 cell transplanted tumor in BALB/c nude mice [44].

The anticancer activities of ginsenosides are inversely proportional to the number of sugar units in the molecule [10]. For instance, ginsenosides with four or more sugar moieties, which present in white ginseng, with a lower ratio in red ginseng and are absent in black ginseng, such as Rb and Rc, did not exert significant anti-proliferative effects, while Rd (three sugar units) weakly inhibited cancer cells growth [31]. On the other hand, the ginsenosides with one or two sugar residues (present in the highest ratio in black ginseng due to the steaming process), such as ginsenosides Rg_3_ (two sugar units), Rh_2_ (one sugar unit) and Rg_5_ (two sugar units), exhibited promising anticancer activities against a wide array of cancer types in several in vitro, in vivo and clinical studies.

Rg_3_ has been found to be a potent inhibitor of invasion of several tumor cell lines including gallbladder cancer (GBC-SD, Mz-ChA-1 and QBC939 cell lines) [45], rat ascites hepatoma (MM1 cell line) [46], melanoma (B16FE7, C8161 and A375 cell lines) [46], human non-small lung carcinoma (H1650, H520 and H1963 cell lines) [47] and human pancreatic adenocarcinoma (PSN-1 cell line) [46], as well as human breast cancer (MDA-MB-231 and MCF-7 cell lines), human liver cancer (HepG2, Hep3B, Hep1-6 and SMMC-7721cell lines) and human colon cancer (HT-29, HCT116, SW480 and HCT116 cell lines) [48].

The anticancer potential of Rg_3_ has been associated with apoptosis induction which was evidenced in HepG2 cells through induction of calcium-dependent apoptosis [49] and downregulation of the hypoxia-responsive transcription factor (HIF-1α) expression [50]. In addition, apoptosis was confirmed in ovarian cancer cell lines via the reduction of (HIF-1α) expression [51] and the downregulation of the Phosphatidylinositol-3 kinases (PI3K)/Akt pathway [52]. Another important mechanism is the inhibition of tumor cells angiogenesis, which have been correlated mostly with the inhibition of the vascular endothelial growth factor (VEGF) expression in different human cancer cell lines such as esophageal carcinoma [53] and Lewis lung carcinoma [54].

The cytotoxic effects of Rg_3_ were also exerted as a result of the induction of DNA double-strand breaks in human osteosarcoma cell lines (MG-63, OS732, U-2OS and HOS) [55] and through the induction of cell detachment and modulation of MAP kinases in prostate cancer cell lines (LNCaP and PC3) with EC_50_ values of 8.4 and 14.1 μM, respectively [56]. In human glioma cells, Rg_3_ induced senescence-like growth arrest by regulating Akt and p53/p21-dependent signaling pathways [57] and altered cellular redox state in opposite directions by increasing the cellular GSH/GSSG ratio, enhancing the γ-GCS activity and suppressing ROS generation [58], while in human colon cancer cell lines it inhibited micro-lymphatic metastasis [59] and blocked the nuclear translocation by reason of downregulation of Wnt/β-catenin signaling [60]. Human melanoma cell lines have been inhibited by Rg_3_ through the down-regulation of histone deacetylase 3 (HDAC3) and the up-regulation of p53 acetylation [61] in addition to the decrease of fucosyltransferase IV (FUT4) and its synthetic product Lewis Y (LeY) FUT4/LeY expression and the inhibition of EGFR/MAPK pathway activation [62].

The in vivo anticancer activities of Rg_3_ have been examined in several experiments; it could inhibit tumor growth and metastasis of human gastric cancer in SCID mice as it decreased intratumoral microvessel density [63] and reduced lung tumor incidence in newborn mice injected with benzo(a)pyrene [64]. Likewise, it reversed multidrug resistance (MDR) of lung adenocarcinoma in mice via the downregulation of MDR-mediated proteins, P-glycoprotein (P-gp), multidrug resistance-associated protein (MPR1) and lung resistance protein 1 (LPR1) [65]. In hepatocellular carcinoma H22-bearing mice, both isomers of Rg_3_, 20(S) and 20(R), improved cellular immunity, stimulated conA-induced lymphocyte proliferation and augmented Th1-type cytokines IL-2 and IFN-γ levels, with a notice that 20(R)-Rg_3_ exhibited stronger activities [66].

The synergetic effect of Rg_3_ with different anticancer agents has been verified in different studies with several drugs, such as paclitaxel [67], docetaxel [68], 5-fluorouracil (5-FU) [69], doxorubicin [70], arsenic trioxide (As_2_O_3_) [71], capecitabine [72], cisplatin [73], gemcitabine [74], mitomycin C and tegafur [75]. ‘Shen-Yi capsule’ is a commercial product of ginsenoside Rg_3_ distributed in the Chinese pharmaceutical market as an anticancer drug and used mostly in combination with several other chemotherapeutic agents [76].

The ginsenoside Rh_2_ could suppress proliferation in several cancer cells, including human breast cancer (MCF-7) [77], lung cancer adenocarcinoma (A549 cells) [78], human colorectal carcinoma (HCT116) [79], prostate cancer (LNCaP and PC3) [56]. Leukemia (HL-60) [80], uterine leiomyoma [81], neuroblastoma (SK-N-BE-2) [82], glioblastoma multiforme (GBM) [83], human gastric cancer (SGC-7901) [84], hepatocellular carcinoma (HPEG-2) [85], intestinal (Int-407 and Caco-2) [86] and mouse melanoma (B16) [87].

The antiproliferative effect of Rh_2_ appears to be linked to its ability to induce apoptosis in cancer cells and arrest cell cycle progression. For instance, Rh_2_ has been reported to affect the regulation of caspase enzymes, major proenzymes affecting apoptosis, in prostate cancer cells [56], human neuroblastoma SK-N-BE(2) [82] and human lung adenocarcinoma A549 cells [15]. Rh_2_ could induce calcium-dependent apoptosis and autophagy in HepG2 cells [49] and cause cell cycle arrest at the G1 stage in human lung adenocarcinoma A549 cells [88], MCF-7 human breast cancer cells and SK-HEP-1 hepatoma cells [11].

Rh_2_ was also able toinhibit cancer cells angiogenesis via the inhibition of vascular endothelial growth factor A (VEGF-A) protein in GBM cells [83], induce cell detachment and modulate mitogen-activated protein (MAP) kinases in the prostate cancer cell lines, LNCaP and PC3 [56], in addition to hepatic cellular carcinoma HepG2 cells [10]. The inhibitory effects of Rh_2_ against the growth of glioblastoma and hepatocellular carcinoma in vitro and in vivo in a mouse model have been outlined [89,90]; these effects were associated with a significant increase in apoptosis induction and decrease in proliferation of tumor cell through the inhibition of the epidermal growth factor receptor (EGFR) signaling pathway. In another in vivo study, Rh_2_ could reduce lung tumor incidence in newborn mice after injection with benzo(a)pyrene [64]. Likewise, Rh_2_ showed anti-proliferative ability against prostatic cancer cells invasiveness in vivo and in vitro via the activation of transforming growth factor β (TGFβ) receptor signaling [91].

The dose-dependent significant inhibition of Rg_5_ against basic fibroblast growth factor (bFGF)-induced endothelial cell proliferation and migration has been revealed [42]. Rg_5_ also could arrest the cell cycle of human hepatoma SK-HEP-1 cells via the down-regulation of cyclin E-dependent kinase activity [92] and of human breast cancer (MCF-7) in G0/G1 phase through the regulation of cell cycle-related proteins and the regulation of the expression of apoptosis-related proteins including Bax, PARP and cytochrome [43]. In another experiment, the ability of Rg_5_ to induce a significant increase in apoptosis induction and DNA damage in five human cervical cancer cell lines (HeLa, MS751, C33A, Me180 and HT-3) has been verified in a time and concentration-dependent manner [93]. Through an in vitro and in vivo study, Rg_5_ controlled chemotherapeutic multidrug resistance (MDR) mediated by ABCB1 transporter via the increase of the accumulation of ABCB1 substrates intracellularly [93]. In addition, the combination of Rg_5_ and docetaxel (TXT) in nude mice bearing an A549/T tumor could suppress the growth of drug-resistant tumors significantly through the suppression of AKT phosphorylation and Nrf2 expression [94].

Compound K exhibited promising inhibitory activities against several types of cancer cell lines including lung carcinoma (B16-BL6 [95] and 95-D [96]), leukemia (HL-60 [97], K562 [96], Kasumi-1 and MV4-11 [98]), hepatoma (HepG2 [99], HGC-27 [100] and SMMC7721 [101]), colorectal cancer (colon 205 [100], HCT-116, SW-480 and HT-29 [102]), gastric carcinoma (MKN-45 [98], BGC823 and SGC7901 [103]), breast cancer (MCF-7 [104]) pulmonary adenocarcinoma (PC-14 [97]), nasopharyngeal carcinoma (HK-1 [105]), prostate cancer (Du145 [100]) and brain tumors (human astroglioma U87MG, CRT-MG and U373MG [106]).

In addition, compound K significantly inhibited the growth and metastasis formation of glioblastoma U87MG and U373MG cell lines through several mechanisms, including cell cycle arrest at the G0/G1 phase, decreasing the expression levels of cyclin D1 and cyclin D3, apoptosis induction via nuclear condensation, increasing in ROS generation, mitochondrial membrane potential depolarization and activation of caspase-3, caspase-9 and poly(ADP-ribose) polymerase (PARP) enzymes [107]. Furthermore, compound K showed promising in vivo anticancer activities against many cancer types; the ability of compound K to induce apoptosis through the loss of mitochondrial membrane potential and activation of caspase 3 in lung cancer of nude mice has been reported [10]. In another study, compound K significantly inhibited metastasis induced by IV injection of B16-BL6 lung melanoma cells in syngeneic mice [108]; it also exhibited inhibitory activity against colon cancer in an in vivo study via several pathways, including antiproliferation, apoptosis induction and cell cycle arrest in the G1 phase [102].

The significant inhibition of the acidic polysaccharide (Ginsan) for benzo[a]pyrene-induced autochthonous lung tumors in mice through the induction of Th1 cell and macrophage cytokines has been outlined [109]. PGP2a is an acidic protein–polysaccharide with a molecular weight of 3.2 × 10^4^ Da and consists of galactose, arabinose, glucose and galacturonic acid in the molar ratio of 3.7:1.6:0.5:5.4, respectively. PGP2a inhibited the growth of human gastric cancer HGC-27 cells dose-dependently through apoptosis induction and cell cycle arrest in G2/M phase; PGP2a suppressed the protein expression of Twist and AKR1C2, with an increase of NF1 [110].

### 4.2. Immunomodulatory and Anti-Inflammatory Effects

In a comparative study, black ginseng exhibited stronger anti-inflammatory and anti-nociceptive effects than red ginseng in xylene-induced ear edema model in mice and carrageenan-induced paw edema in rats; it inhibited the pro-inflammatory mediators, inducible nitric oxide synthase (iNOS) and cyclooxygenase-2 (COX-2) and pro-inflammatory cytokines, IL-1β, interleukin-6 (IL-6) and tumor necrosis factor-α (TNF-α) [111]. In addition, the lipopolysaccharide-induced TNF-α release was significantly decreased after treatment with black ginseng extract [41]. Furthermore, the dose-dependent recovering effect of black ginseng extract against the cisplatin-induced nephrotoxicity and the reduced (pig cell LLC-PK1) cells viability, has been indicated [112]. In addition, in the same experiment, compound K could abrogate the elevated percentage of apoptotic LLC-PK1 cells significantly.

The black ginseng extract potently inhibited atopic dermatitis and asthma through suppression of the elevated IL-6 and IL-8 induced by *Dermatophagoides pteronissinus* treatment in human acute monocytic leukemia (THP-1) and human eosinophilic leukemic (EoL-1) cell lines [113]. Similarly, gamma-irradiated black ginseng extract could inhibit mast cell degranulation and suppress atopic dermatitis-like skin lesions in mice through different mechanisms, including the suppression of β-hexosaminidase and histamine in the stimulated mucosal mast cells [114].

In another study, the significant inhibitory effect of pretreatment of dorsal skins of female ICR mice with Rg_3_ against 12-O-tetradecanoylphorbol-13-acetate (TPA)-induced ornithine decarboxylase activity and 7,12-dimethylbenz[a]anthracene-initiated papilloma formation was declared [15]. Rg_3_ could inhibit COX-2 expression and eukaryotic transcription factor, NF-kappaB activation. Rg_3_ could suppress the nitric oxide (NO), reactive oxygen species (ROS) and prostaglandin E2 (PGE2) productions induced by lipopolysaccharide (LPS) in RAW264.7 macrophage cells dose-dependently [115].

The ginsenoside Rh_2_ inhibited the production of NO, with an IC_50_ value of 17 μM in LPS/interferon-γ-stimulated BV-2 microglial cells. This effect correlated with the inhibition of the expression of COX-2 and pro-inflammatory TNF-α, IL-1β, while it increased the expression of the anti-inflammatory cytokine IL-10 [116]. Compound K exhibited anti-inflammatory activities in mice against several kinds of inflammations such as carrageenan-induced paw edema, colitic (DSS and TNBS-induced), sepsis (zymosan and LPS-induced) and xylene-induced ear edema through the inhibition of the activation of ROS, MAPKs and NF-κB/AP-1 with the enhancement of HO-1/ARE signaling [108]. Compound K significantly increased the inflammatory pain threshold, reduced PGE2 level and decreased COX-2 expression in rats [108]. In LPS-activated RAW264.7 cells, compound K inhibited the expression of the proinflammatory cytokines with a concentration of 5 μM; it also reduced the expression of other inflammatory mediators such as IL-1β, COX-2, iNOS and TNF-α through the mediation of NF-κB [15]. Both of Rh_2_ and compound K significantly inhibited the passive cutaneous anaphylaxis (PCA) reaction induced by IgE in rodents through membrane stabilizing effect [11].

RGAP is an acidic polysaccharide of ginseng containing 56.9% acidic sugars and 28.3% neutral sugars. The intraperitoneal administration of RGAP exerted promising in vivo and in vitro immunomodulating activities through regulation of NO synthesis in female BALB/c mice [117]. RGAP could also augment the humoral immune response of pidotimod in immunosuppressed mice in response to both lipopolysaccharide and sheep red blood cells through increasing the number of plaque-forming cells in the spleen [118].

Ascorbic acid, cinnamic acid and esculetin were found to be the most abundant phenolics in *Panax ginseng*; ascorbic acid and cinnamic acid effectively suppressed LPS-induced nitric oxide production in the RAW 264.7 cells; in addition, cinnamic acid could significantly inhibit the oxidative damage in the human neuroblastoma SH-SY5Y cells [119].

### 4.3. Hepatoprotective Effect

Black ginseng exhibited a hepatoprotective effect on acetaminophen-induced mice liver injury decreasing the levels of serum alanine aminotransferase (ALT), aspartate transaminase (AST) and lipid peroxidation product malondialdehyde (MDA) significantly. Meanwhile, the antioxidant levels in liver tissues were elevated, including glutathione (GSH), cytochrome P450 E1 (CYP2E1) and 4- hydroxynonenal (4-HNE) [120]. Additionally, treatment with black ginseng ethanol extract decreased lipid accumulation in the liver and damage in the muscle of diabetic mice through the activation of AMP-activated protein kinase (AMPK) [121]. Symmetrically, black ginseng could decrease the activation of apoptotic pathways in the liver through decreasing Bax and increasing Bcl-2 protein expression levels associating a significant inhibition of APAP-induced necrosis and inflammatory infiltration [120]. Fermented black ginseng exhibited hepatoprotective activities against hydrogen peroxide (H_2_O_2_)-mediated oxidative stress in HepG2 human hepatocellular carcinoma cell lines. It could attenuate the increased ROS level and elevate the expression and activity of antioxidant enzymes, such as superoxide dismutase, catalase and glutathione peroxidase. At the same time, black ginseng inhibited the phosphorylation of upstream mitogen-activated protein kinases (MAPKs) [23]. Oral administration of the ginsenosides Rg_3_, Rh_2_ and compound K, in addition to intraperitoneal administration of Rh_2_ and compound K, significantly inhibited the increase of serum ALT and AST levels in tert-Butyl Hydroperoxide (t-BHP)-liver damage-induced mice [122,123].

### 4.4. Antidiabetic Effect

In a comparative experiment, black ginseng ethanolic extract exerted stronger antidiabetic activity than red ginseng. It reduced hyperglycemia at a dose of 200 mg/kg in STZ-treated mice and improved the β-cell function by inhibition of β-cell apoptosis through suppression of the cytokine-induced nuclear factor–κB signaling pathway in the pancreas [124]. Black ginseng extract decreased the elevated blood glucose levels in streptozotocin-induced diabetic rats to a normal level [125]. Additionally, black ginseng ethanol extract decreased fasting blood sugar and glycated hemoglobin (HbA1c) via the upregulation of GLUT2 and GLUT4 expression in diabetic rats [121]. In another experiment, black ginseng ethanolic extract could significantly improve fasting blood glucose levels, glucose tolerance and decrease HbA1c in STZ-induced diabetic mice. It could increase glucose uptake in C2C12 myotubes via AMPK, Sirt1 and PI3-K pathways. In addition, a significant increase in the expression of the genes involved in glucose uptake in the muscle (glucose transporter (GLUT)1, GLUT4) and β-oxidation (acyl-CoA oxidase (ACO), carnitine palmitoyl transferase 1a (CPT1a), mitochondrial medium-chain acyl-CoA dehydrogenase (MCAD)) has been reported [126].

The ginsenoside Rh_2_ could increase insulin secretion and decrease plasma glucose level in Wistar rats through the increase of acetylcholine release from nerve terminals stimulating the muscarinic M_3_ receptors in pancreatic cells [127]. Rh_2_ could reverse the impaired β-cell growth potential and inhibit apoptosis tendency via regulation of cell cycle proteins and modulation of Akt/Foxo1/PDX-1 signaling pathway [128]. In a similar experiment, Rg_3_ enhanced islet cell function and attenuated cytokine-induced damage associated with NO production and apoptosis in mouse islets [129]. Oral dosage 25 mg/kg of compound K could lower the plasma glucose, TG, cholesterol and NEFA levels by 20.7%, 41.6%, 20.2%, and 24.6%, respectively [108].

### 4.5. Anti-Obesity and Antihyperlipidemic Effects

Black ginseng ethanol extract could decrease the induced hyperlipidemia and fat accumulation in white adipose tissues and liver in the fat diet-mice through the inhibition of fat digestion [130,131]. Black ginseng extract efficiently reduced the total serum cholesterol levels and low-density lipoprotein (LDL) levels in the fat diet-fed mice [131] and in STZ-induced diabetic mice [126]. In addition, a significant decrease in triglyceride and non-esterified fatty acid (NEFA) levels with an increase in HDL level was noticed in male obese diabetic C57BLKS/J-db/db mice after treatment with black ginseng [132]. Water and ethanol extracts of black ginseng decreased lipid accumulation through the regulation of PPARγ, C/EBPα and AMPK phosphorylation in 3T3-L1 cells with a stronger activity for the ethanol extract [133]. Black ginseng could regulate the expression of hepatic genes involved in gluconeogenesis (phosphoenol pyruvate carboxykinase (PEPCK), glucose6 phosphatase (G6Pase)), glycogenolysis (liver glycogen phosphorylase (LGP)) and glycogenesis (glycogen synthase (GS)) [126] and attenuate the key genes responsible for lipogenesis (acetyl-coenzyme A (CoA) acetyltransferase, 2, 3-hydroxy-3-methyl-glutaryl-CoA reductase) [131].

Oral dosage of 25 mg/kg of compound K in tert-butylhydroperoxide (t-BHP)-induced liver injured mice, could inhibit the increase of serum ALT and AST levels to 13.2% and 8.3%, respectively, while the intraperitoneal administration with a dosage of 10 mg/kg inhibited serum ALT and AST levels to 3.3% and 42%, respectively [108].

### 4.6. Effects on the Central Nervous System

Black ginseng extract could prevent the cognitive impairment induced by cholinergic dysfunction through the inhibition of acetylcholinesterase (AChE) activity after 24 h of a single administration of 200 mg/kg in the brain [21]. In a comparative experiment, both white ginseng and black ginseng of the roots of *Panax ginseng*, *P. quinquefolium* and *P. notoginseng* inhibited AChE and butyrylcholinesterase (BChE) dose-dependently. The efficacy of black ginseng roots was greater than that of the respective white ginseng roots of each species [33]. Black ginseng extract significantly reversed scopolamine (SCOP)-induced memory impairment in amnesic mice and also reduced escape latency by decreasing malondialdehyde (MDA) levels and restored superoxide dismutase (SOD) and catalase (CAT) activities [134]. The same effect was confirmed by the black ginseng-enriched formula (Chong–Myung–Tang) in the water maze associating the inhibition of nitric oxide production in BV2 cells and significant suppression of expression of proinflammatory cytokines such as nitric oxide synthase, cyclooxygenase-2 and interleukin-1b. Over and above, the black ginseng-enriched CMT extract diminished the protein expression of MAP kinase and NF-kB pathway factors [135].

Likewise, black ginseng protected rats against ischemia-induced neuronal and cognitive impairment and improved the escape latency with reduced loss of cholinergic immunoreactivity and nicotinamide adenine dinucleotide phosphate-diaphorase (NADPH-d)-positive neurons in the hippocampus [136]. After oral administration of black ginseng extract with a dose of 200 mg/kg for 16 weeks, the cognitive deficits associated with normal aging in old-age mice was decreased, associating DNA damage protection and a significant increase in brain-derived neurotrophic factor protein expression [137].

The ginsenosides Rh_2_ and Rg_3_ exhibited treating effects against neurodegenerative disorders by showing inhibitory effects in cultured hippocampal neurons. The additive effects of Rg_3_ and Rh_2_ on N Methyl Dextro Aspartic Acid (NMDA) receptors suggested that they modulate different NMDA receptor regulatory sites [138,139]. Ginsenoside Rh_2_ could significantly improve learning and memory performance and reduce brain senile plaques at 14-month-old model mice through the reduction of amyloid beta (Aβ) secretion and amyloid precursor protein (APP) endocytosis [15].

Further to this, sublingual vein injection of the ginsenoside Rg_3_ at a dose of 5 mg/kg exhibited significant neuroprotective effects on rats against focal cerebral ischemic injury associated with a decrease in neurological deficit scores, reduction of the infarct area and enhancement of the cerebral blood flow. At the same time, Rg_3_ could significantly improve mitochondrial energy metabolism, antagonize the decreases in GSH-Px and SOD activities and increase in MDA level induced by cerebral ischemia, suggesting that Rg_3_ works via the reduction of lipid peroxides, scavenging free radicals and improving the energy metabolism [140].

In another study, Rg_5_ could improve cognitive dysfunction and attenuated neuroinflammatory responses in STZ-induced memory-impaired rats dose-dependently together with decreased levels of inflammatory cytokines TNF-α, IL-1β and (AChE) activity and a high increase in choline acetyltransferase (ChAT) activity. Rg_5_ alleviated Aβ deposition but enhanced the expressions of insulin-like growth factor 1 (IGF-1) and brain-derived neurophic factor (BDNF) in the hippocampus and cerebral cortex and downregulated the elevated expressions of COX-2 and iNOS [141].

### 4.7. Antioxidant Effect

There was an increase in the content of total phenolics because of steaming. This increase is thought to be a direct reason for the higher antioxidant activity of black ginseng [142]. Black ginseng roots of *Panax ginseng*, *P. quinquefolium* and *P. notoginseng* exhibited more total phenolic contents and 2,2-diphenyl-1-picryl-hydrazyl (DPPH) scavenging activity than white corresponding roots. The best antioxidant activity was obtained with black ginseng roots of *P. ginseng* [33]. The same result was confirmed by another experiment through improving antioxidant activity according to the increasing number of steaming–drying cycles [143]. Black ginseng improved most of the morphological scores significantly in ethanol-treated embryos associating the significant restoration of the decreased mRNA levels of the antioxidant enzymes; cytosolic glutathione peroxidase (GPx), phospholipid hydroperoxide GPx and selenoprotein P, suggesting that black ginseng exerted this protective effect via the augmentation of antioxidative effect in the embryo [144]. Unexpectedly, in an acetaminophen-induced oxidative stress rat model, it was found that red ginseng extract exhibited more anti-oxidant activity than white ginseng and black ginseng extracts [145].

Both Rg_3_ and Rh_2_ reduced the generation of ROS, which was induced by ethanol in mouse hepatocyte cells, while in another experiment, Rg_3_ could significantly inhibit cyclophosphamide-induced oxidative stress through the upregulation of lysozyme, catalase and SODs activities in addition to the reduction of the levels of malondialdehyde and nitric oxide in several organs [15]

In addition, the increased Maillard reaction products as a result of steaming and drying are major contributors to a stronger antioxidant effect [34]. Maltol inhibited Fe^2+^-stimulated DNA degradation by bleomycin with little prooxidant activity; it might act as a scavenger of free radicals as through its in vitro ability to inhibit erythrocyte membrane protein polymerization and membrane lipids oxidization after exposure to butyl hydroperoxide [146]. Four acidic polysaccharide fractions (BGP-60, BGP-65, BGP-70 and BGP-80) with estimated molecular weights of 28.6, 26.7, 11.4 and 3.05 kDa, respectively, were purified from black ginseng and showed strong potential antioxidant activities against DPPH, superoxide anion radicals and hydroxyl radical [147].

### 4.8. Tonic Effect

Black ginseng treatments at a dose of 150 mg/kg significantly increased the exercise capacity in rats. The level of blood lactic acid was decreased but the activity of citrate synthase in muscles was increased [148]. Black ginseng also increased muscle growth and could treat or prevent muscle loss related to aging through the production of myoblasts with larger multinucleated myotubes and increased diameter and thickness; the mechanism of action is thought to be the activation of Akt/mTOR/p70S6k axis [149]. The complex extract of black ginseng and fenugreek could increase cell viability, which had been attenuated because of oxidative stress through regulation of Erk kinase activation. Moreover, the oral administration of the complex could significantly increase the levels of total and bioavailable testosterone, follicle-stimulating hormone and luteinizing hormone in a hormone-deficient animal model. A dosage of 100 mg/kg of the complex extract could improve motor function and increase muscle endurance in a forced swimming test [150]. In a comparative study, the acidic polysaccharide of black ginseng exhibited a stronger anti-fatigue activity, than the neutral polysaccharide using the forced swim test through the restoring of the physiological markers for fatigue including glucose, glutathione peroxidase (GPx), creatine phosphokinase (CK), lactic dehydrogenase (LDH) and malondialdehyde (MDA) levels [151].

### 4.9. Topical Uses

Fermented black ginseng exhibited a significant anti-wrinkle effect at a concentration of 0.3 μg/mL through the increase of the type I procollagen expression levels in the human fibroblasts and the decrease in the MMP-1 expression level. Furthermore, at 3 μg/mL it increased the expression of TIMP-2 up to 154.55%. However, at 10 μg/mL it decreased the expression levels of MMP-2 and MMP-9 to 45.15% and 66.65%, respectively [152].

The wound healing activity of fermented black ginseng in human umbilical vein endothelial cells was mediated by angiogenesis through the MAP kinase pathway and enhanced the tube formation in HUVECs and migration in HaCaT cells was discussed [153]. Fermented black ginseng could stimulate the phosphorylation of p38 and extracellular signal-regulated kinase in HaCaT cells. Moreover, mice treated with 25 mg/mL exhibited faster wound closure in the experimental cutaneous wounds model [153].

The ginsenosides Rg_5_ and Rk_1_ exhibited skin-whitening efficacies in vitro as well as in vivo using human skin and zebrafish embryos. The inhibition of melanin activity and the decrease in tyrosinase levels was confirmed through the activation of the MEK-ERK signaling pathway [154].

### 4.10. Toxicity Studies

Single acute oral toxicity of black ginseng has been studied in rats indicating that the oral LD_50_ in the rats is higher than 15 g/kg, meaning that the black ginseng is virtually nontoxic [155]. On a cellular level, black ginseng showed no significant cytotoxicity against the normal splenocyte cells [41], while RG_3_ protected normal cells against cancer by reduction of MNNG-induced DNA damage and apoptosis [55]. The eye irritation potential of fermented black ginseng was examined using the EpiOcular-EIT kit; results indicated that black ginseng considered is safe for the eyes at concentrations up to 100 μg/mL [152]. Fermented black ginseng could ameliorate the nephrotoxicity via regulating oxidative stress, inflammation and apoptosis with a DPPH radical scavenging activity stronger than that of white ginseng. Moreover, the reduced creatinine clearance levels and cell viability by cisplatin were recovered significantly after treatment [156]. Similarly, black ginseng extract and Rg_3_ could recover the cisplatin-induced nephrotoxicity and the reduced (pig cell LLC-PK1) cells viability dose-dependently [112].

## 5. Methodology

A systematic literature search of several online databases, such as NCBI, Scopus, Science Direct, PubMed and google scholar, was conducted up to April 2019. The principal search topics were related to black ginseng: Its preparation methods, transformed ginsenosides and phytochemical changes in black ginseng in addition to the pharmacological effects of black ginseng, ginsenosides and other major secondary metabolites. The main sources of data collection included review articles and research papers published by reputed publishers such as Elsevier, Springer, Routledge and Taylor & Francis.

### Prospects

Ginseng was first discovered and used by ancient Chinese. Traditionally, only white ginseng (or sun-dried ginseng), red ginseng and sugar ginseng were used as food and drugs, but because of the big loss of ginsenosides in the sugar ginseng, it is rarely used nowadays. Black ginseng is a newly-processed ginseng product with much stronger secondary metabolites; here are three prospects proposed to peers:

While black ginseng is a newly processed ginseng, there are several products made of it and which have been approved in the health and food markets with a large consumption in the rich areas of China and Korea. Accordingly, further deep studies are imperative and safety assessment is required. Presently, ginseng aged less than 5 years could be used as a food material in China with a dose of 3g/day at maximum. Similarly, and because of the stronger anticancer activity of black ginseng, its dose limit should be precisely issued by the China Food and Drug Administration (CFDA) in China and other countries.

Unfortunately, almost all the scientific studies of black ginseng examined it as one drug, not as a member in a formula like most of the drugs in traditional Chinese medicine. Is there another medicine that could or could not be mixed with black ginseng or should it be used as one treatment? In addition, if the black ginseng could be used in a formula, the determination of its proper dose inside the formula needs further study. Furthermore, despite there being several clinical comparative studies of the three types of ginseng (white ginseng, red ginseng and black ginseng), the anti-fatigue action associated with qi deficiency—which is a vital action of ginseng—has not been studied sufficiently.

More research should be driven toward the transformed ginsenosides of black ginseng; some of these ginsenosides have been detected in wild ginseng, such as Rg_3_ and Rh_2_ [157]; the engagement of these rare ginsenosides may elucidate the medicinal value of wild ginseng, which was a highly esteemed part of the old Chinese culture until now.. Additionally, it has been proven that the primary saponins usually biotransform in humans’ digestive tracts into the more active transformed saponins and/or their aglycone [10]. Presently, the ginsenoside Rg_3_ is developed as a cheap anticancer auxiliary medicine in China. The establishment of a simple preparation procedure of black ginseng decreased the preparation cost, which gives a good chance to develop a cheap and effective method to prepare these valuable transformed ginsenosides.

In summary, research in black ginseng revealed a massive change in the phytochemical content aligned with the priority in several pharmacological actions of white ginseng and red ginseng; consequently, black ginseng is expected to play a greater role in the health of humankind as the research goes on.
